# Magnetic bead-assisted endoscopic submucosal dissection: a gravity-based traction method for treating large superficial colorectal tumors

**DOI:** 10.1007/s00464-019-06799-7

**Published:** 2019-04-24

**Authors:** Liansong Ye, Xianglei Yuan, Maoyin Pang, Johannes Bethge, Mark Ellrichmann, Jiang Du, Xianhui Zeng, Chengwei Tang, Stefan Schreiber, Bing Hu

**Affiliations:** 1 Department of Gastroenterology, West China Hospital, Sichuan University, No. 37, Guo Xue Alley, Wu Hou District, Chengdu, 610041 Sichuan China; 2 Department of Gastroenterology, Georgetown University Hospital, Washington, DC USA; 3 Department of Gastroenterology, University Medical Center Schleswig Holstein, Campus Kiel, Kiel, Germany

**Keywords:** Colorectal tumor, Endoscopic submucosal dissection, Traction, Gravity, Magnet

## Abstract

**Background:**

Colorectal endoscopic submucosal dissection (ESD) has always been challenging for endoscopists, but the procedure can be facilitated after adequate exposure of submucosal layer and cutting line. We developed a traction method based on gravity for facilitating colorectal ESD, referred as magnetic bead-assisted ESD (MBA-ESD). This study aimed to compare the safety and effectiveness of MBA-ESD and conventional ESD for treating large superficial colorectal tumors.

**Methods:**

This retrospective study included consecutive patients with large (≥ 20 mm in their maximal diameter) superficial colorectal tumors who underwent MBA-ESD or conventional ESD at our endoscopy center between June 2017 to January 2018. Each patient in the MBA-ESD group was matched to a patient in the conventional ESD group using propensity scores.

**Results:**

Thirteen patients in each group were matched for the analyses. The baseline characteristics were balanced after propensity matching. The incidence of overall complications was significantly lower in the matched MBA-ESD group (0% vs. 38.5%, *P *= 0.039), while similar rates of en bloc resection, R0 resection, curative resection, and tumor recurrence were noted. Although without statistic difference, dissection time and speed were improved when using MBA-ESD (33 min vs. 40 min, *P *= 0.111; and 21 mm^2^/min vs. 16 mm^2^/min, *P *= 0.143, respectively).

**Conclusions:**

MBA-ESD is a feasible, safe, and effective method for treating large superficial colorectal tumors. Further large, prospective and controlled studies are needed to fully assess this method.

Endoscopic submucosal dissection (ESD) has been considered as one of the main options for en bloc resection of large superficial gastrointestinal tumors [1], but colorectal ESD keeps technically challenging for endoscopists around the world [2–4], that is attributed to the unfavorable characteristics of thin wall and angulated lumen in colorectum. The key of safe ESD is to adequately expose the submucosal layer and the cutting line for precise dissection during the whole procedure [5]. Although multiple traction methods have been attempted for submucosal exposure [6], traction by gravity is still one of the most commonly used methods for facilitating colorectal ESD [5]. However, in clinical practices, we found that even after repeated submucosal injections or patients’ position changing, effective exposure of cutting line for precise dissection cannot be achieved in some large or fibrotic colorectal tumors. We have developed a gravity-based traction method: magnetic bead-assisted ESD (MBA-ESD), in which the weight and strength of traction can be easily adjusted by adding one or more magnetic bead systems in the same or different sites of the tumor for adequate exposure of submucosal layer. We had previously reported this method for treating a small flat tumor with severe fibrosis in the descending colon [7], and a lesion with fibrosis in the descending duodenum [8]. This retrospective study was designed to compare the safety and effectiveness of MBA-ESD and conventional colorectal ESD in the treatment of large superficial colorectal tumors. Propensity score-based matching analysis was conducted to reduce bias between two groups.

## Materials and methods

### Study design

This is a single-center, matched cohort study to compare the safety and effectiveness of MBA-ESD and conventional ESD for large (≥ 20 mm in their maximal diameter) superficial colorectal tumors. The consecutive data on patients who underwent colorectal ESD for superficial tumors at the endoscopy center of West China Hospital between June 2017 and January 2018 were retrospectively collected. The study protocol was conducted according to the Helsinki Declaration and was approved by the Biomedical Research Ethics Committee of the West China Hospital of Sichuan University.

### Patients

Patients with superficial colorectal tumors who underwent MBA-ESD or conventional ESD procedure during the study period were included in this study. Small (less than 20 mm in their maximal diameter) superficial colorectal tumors were excluded. Other exclusion criteria were as follows: (1) patient who underwent hybrid endoscopic mucosal resection (EMR)-ESD for colorectal tumor resection; (2) patients who underwent endoscopic submucosal tunnel dissection (ESTD) for rectal tumor resection. Informed consent was obtained from all included patients.

### MBA-ESD technique

MBA-ESD was performed by an experienced endoscopist (Dr. B.H.), who had performed more than 300 colorectal ESD procedures before this study. All procedures were performed using instruments and steps resembling that of conventional ESD procedures, except the application of one or more magnetic bead systems for adequate exposure of submucosal layer and cutting line after partial dissection.

**Fig. 1:**
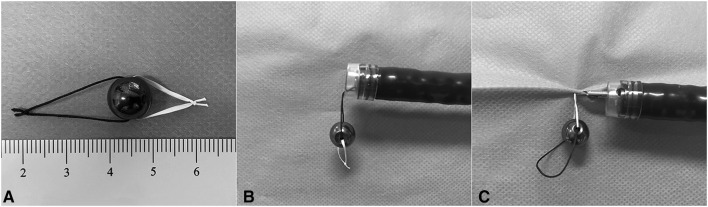
The magnetic bead system and its application diagram. **A** The system consisted of a 1.5-g magnetic bead (10 mm in diameter) and 2 attached strings (20 mm and 10 mm in length, respectively) (two materials of suture and dental floss are used here, but same material with different length can also be selected), **B** the system was brought to tumor location by grasping the long string (suture) using a regular endoclip, **c** the system was applied to the edge of the exfoliated mucosa for weight traction by grasping the short string (dental floss)

**Fig. 2:**
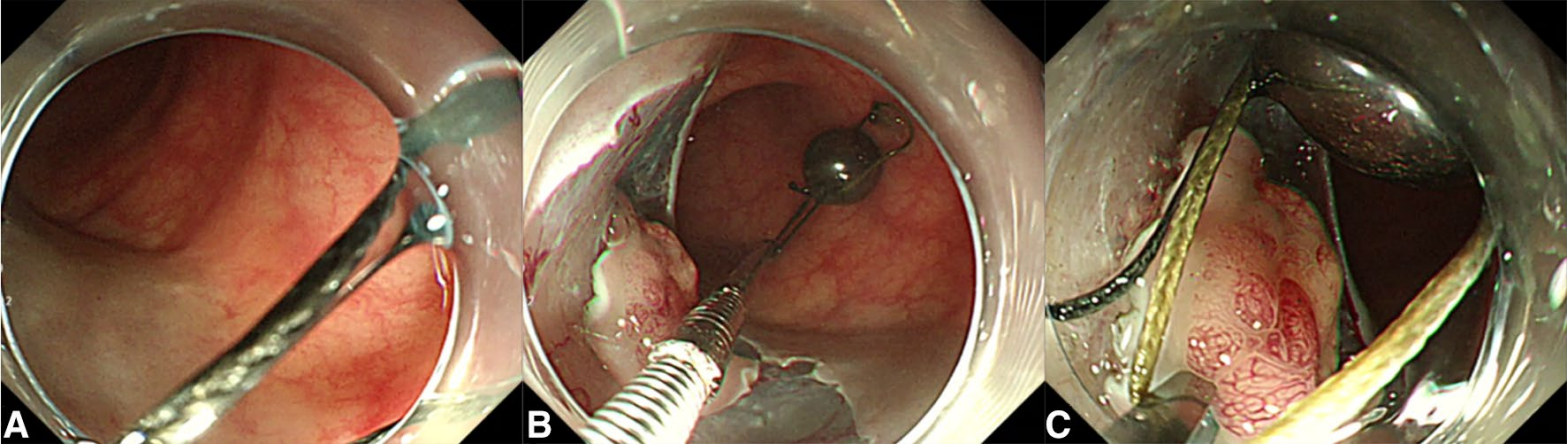
Endoscopic views of the process of insertion and application of the magnetic bead system. **A** Clear endoscopic vision was noted during insertion of the system by clipping the long string, **B** the system was placed in adjacent bowel lumen after arriving at tumor location, **c** the short string was easily grasped by the endoclip for applying to the tumor edge

The system consisted of a magnetic bead (1.5 g in weight and 10 mm in diameter; $0.16/each bead) and 2 attached strings (20 mm and 10 mm in length, respectively; suture and/or dental floss can be selected) (Fig. 1). It was brought to tumor location along with the reintroduction of the endoscope by grasping the 20-mm string using a regular endoclip; the use of such long string helped to avoid the interference of endoscopic vision (Fig. 2). After arriving at the site of tumor, the system was placed at adjacent bowel lumen, allowing a transition of grasping from the long string to the short string. The system was then applied to the edge of exfoliated mucosa by grasping the 10-mm string; this helped to provide effective weight traction without touching the opposite bowel wall. Thus, the submucosal layer and cutting line were adequately exposed for precise dissection. After complete detachment of the tumor, the system was brought out with the specimen using forceps or snare.

**Fig. 3:**
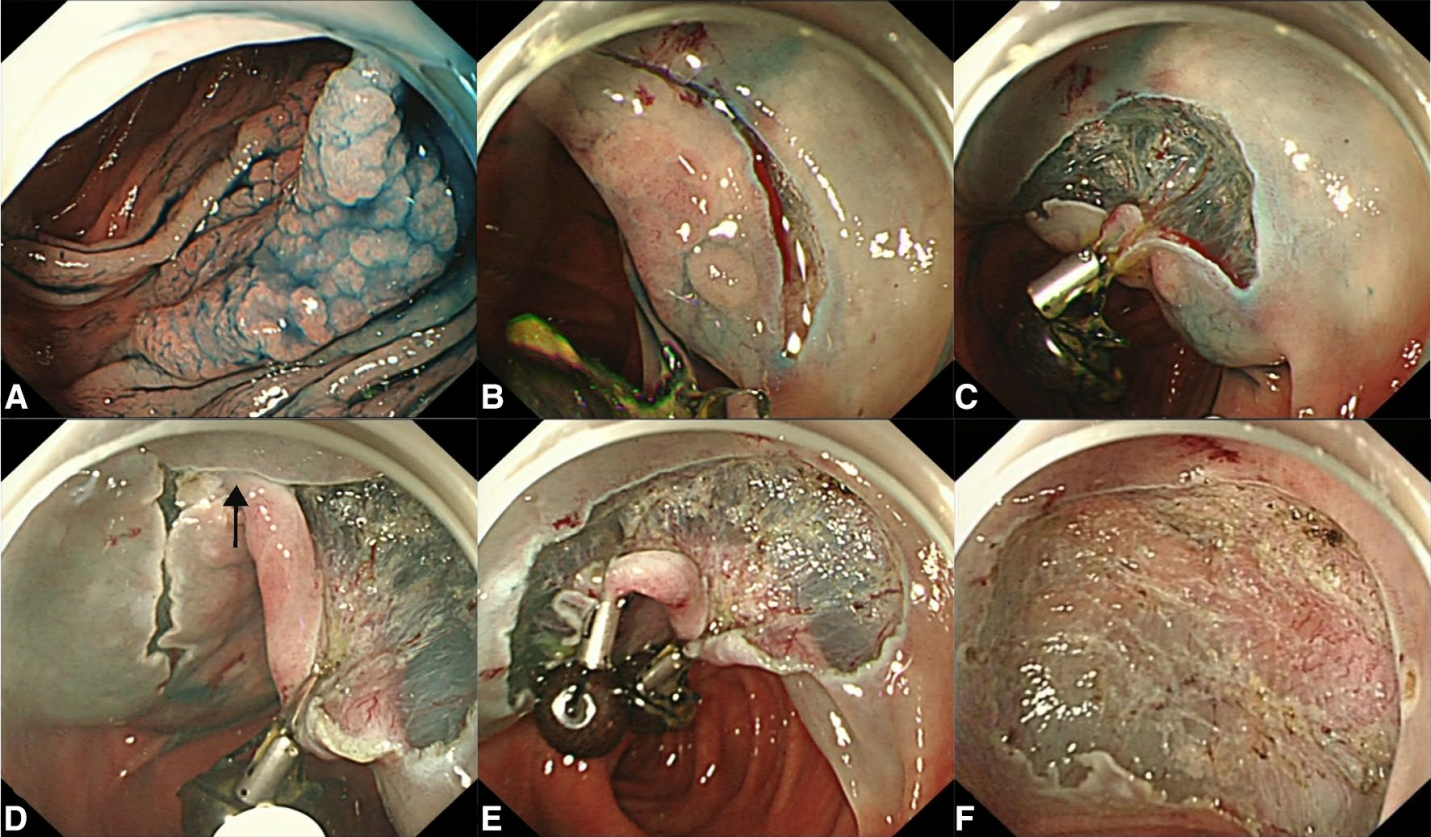
Effective exposure of the submucosal layer achieved by two magnetic bead systems in a large laterally spreading tumor. **A** The large granular lateral spreading tumor in ascending colon, **B** unclear visualization of the submucosal layer noted before traction, **C** adequate submucosal exposure of the local region achieved by applying one magnetic bead system, **D** inadequate submucosal exposure of the adjacent site (arrow) encountered during dissection, **E** adequate submucosal exposure was achieved by adding additional system to adjacent site, **F** the mucosal defect after en bloc resection

The magnetic bead system was used for improving weight traction based on gravity when repeated submucosal injections or patients’ position changing failed to achieve clear submucosal visualization. For tumors with fibrosis, another magnetic bead system can be added to the previously applied system (i.e., the same site) to increase the weight of traction, in which the two magnetic beads coupled together owing to magnetic force [7, 8]. Additional magnetic bead systems, applied to different sites, can also be used for better traction (Fig. 3).

### Outcome measurements

The primary outcome of this study was the rate of complications, including bleeding and perforation occurred during or after the procedure. Immediate bleeding was defined as bleeding occurred during the procedure that needed to be controlled using hemostatic forceps or titanium clips. Delayed bleeding was defined as bleeding symptoms or hemoglobin loss (≥ 2 g/dL) within 30 days after ESD. Immediate perforation was defined as perforation during ESD, and free air detected through radiography. The definition of delayed perforation was perforation after the procedure. Muscularis propria injury was also recorded but was not regarded as a complication.

The secondary outcomes were the rates of en bloc resection, R0 resection, curative resection, and tumor recurrence. Curative resection was defined as previous guideline in 2015 [9]. Tumor recurrence was defined if biopsy samples of follow-up endoscopy revealed the presence of tumor cells.

Other outcomes including dissection time, dissection speed, and specimen integrity were also recorded. Dissection time was measured from submucosal injection to lift surrounding mucosa up to detachment of the tumor, which included the extra time for inserting and applying one or more magnetic bead systems. Dissection speed was defined as area of resected specimen/dissection time (mm^2^/min). The area of tumor was measured by half of the length times half of the width multiplied by 3.14. Specimen integrity was defined as no collateral damage to the specimen, which was associated with traction force and system detachment during the procedure.

### Propensity score matching

Owing to between-group differences in baseline characteristics (especially tumor location and size) in the total cohort, we performed propensity score matching to select patients with 1:1 ratio for both MBA-ESD group and conventional ESD group. The propensity score of undergoing MBA-ESD or conventional ESD was calculated using a multivariable logistic regression model. Since tumor location (rectum, left colon, and right colon), growth type (lateral spreading tumor-granular, lateral spreading tumor-non-granular, and polypoid), and area were three main factors that could affect the conduction of ESD, they were included in the model. Subsequently, each patient in the MBA-ESD group was matched to a patient in the conventional ESD group with the nearest neighbor method using a caliper range of 0.25 of the standard deviation of the pooled propensity scores.

### Statistical analysis

The SPSS 25.0 statistical software was used for analyses and matching. Continual variables were expressed as mean (standard deviation) or median (interquartile ranges) according to their distribution, and were compared using student *T* test or Whitney U-test as appropriate. Categorical variables were expressed as proportion, and were compared using Chi-square test or Fisher’s exact test accordingly. *P* values of ≤ 0.05 were considered significant.

## Results

**Fig. 4:**
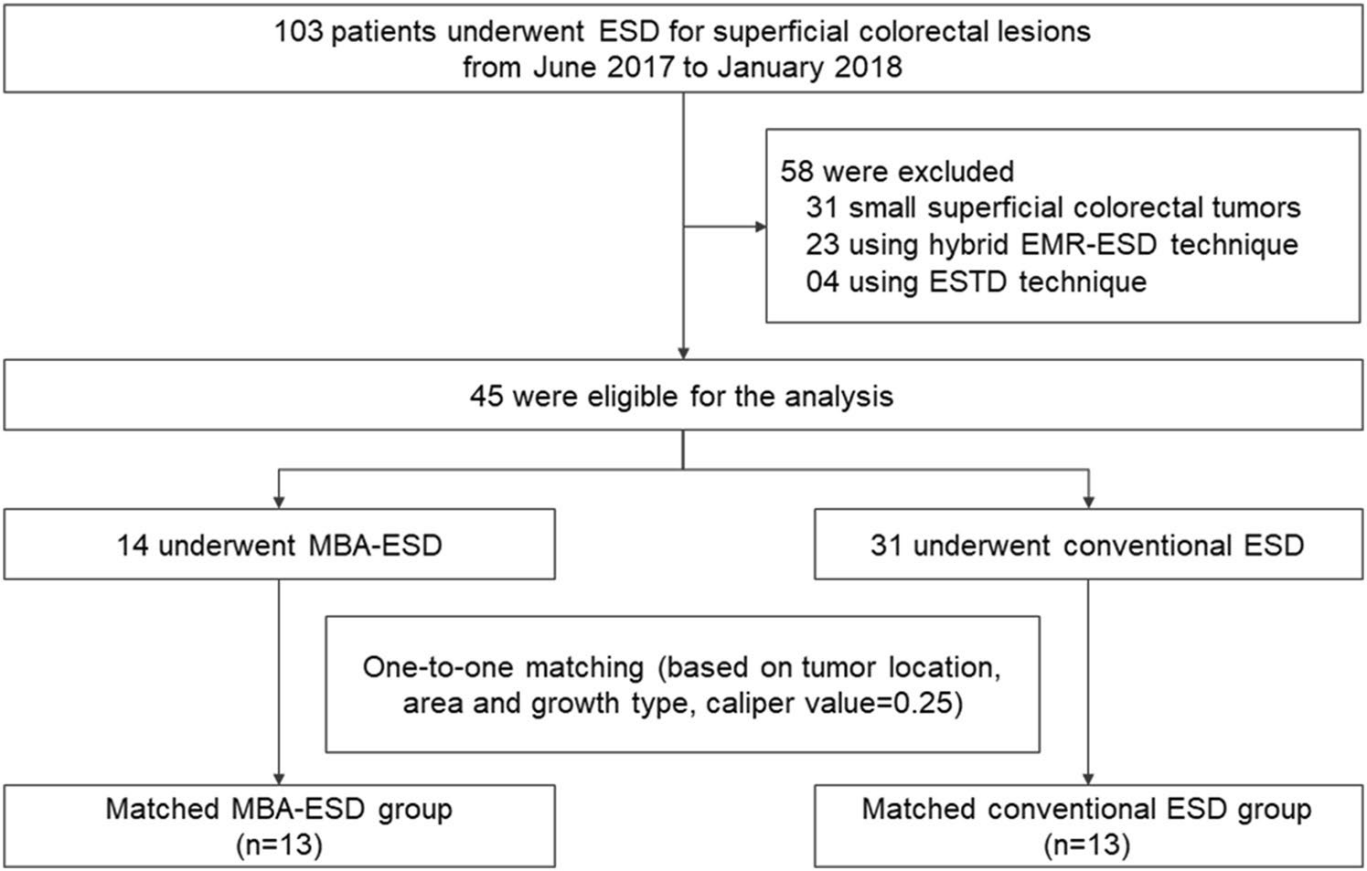
Flowchart of patient selection into the matched groups of MBA-ESD and conventional ESD for large superficial colorectal lesions. *MBA*-*ESD* magnetic bead-assisted endoscopic submucosal dissection, *ESD* endoscopic submucosal dissection, *ESTD* endoscopic submucosal tunnel dissection, *EMR* endoscopic mucosal resection

A total of 103 patients underwent ESD for superficial colorectal tumors from June 2017 to January 2018 (Fig. 4), and 45 patients met the criteria of this study. Among these 45 patients, 14 and 31 patients underwent MBA-ESD and conventional colorectal ESD, respectively. Using the algorithm described above, 13 patients who underwent MBA-ESD were successfully matched to 13 patients who underwent conventional ESD, in which the differences in tumor location, growth type, and area were well balanced (Table 1).

**Table 1:** Baseline characteristics of the total and propensity-matched cohorts for MBA-ESD and conventional ESD for superficial colorectal lesions

	Total cohort			Matched cohort		
	MBA-ESD (*n* = 14)	Conventional ESD (*n* = 31)	*P* value	MBA-ESD (*n* = 13)	Conventional ESD (*n* = 13)	*P* value
Sex			0.492^F^			0.411^F^
Male	11 (78.6%)	20 (65.5%)		10 (76.9%)	7 (53.8%)	
Female	3 (21.4%)	11 (35.5%)		3 (23.1%)	6 (46.2%)	
Age (years^a^)	61 (60–65, 45–70)	63 (53–71, 45–80)	0.667^W^	61 (60–63, 45–70)	64 (52–71, 48–80)	0.354^W^
Location			0.003^F^			0.466^F^
Rectum	2 (14.3%)	19 (61.3%)		2 (15.4%)	5 (38.5%)	
Left colon	3 (21.4%)	7 (22.6%)		3 (23.1%)	3 (23.1%)	
Right colon	9 (64.3%)	5 (16.1%)		8 (61.5%)	5 (38.5%)	
Growth type			0.838^F^			1.000^F^
LST-G	12 (85.7%)	24 (77.4%)		11 (84.6%)	12 (84.6%)	
LST-NG	1 (7.1%)	2 (6.5%)		1 (7.7%)	0 (0%)	
Polypoid	1 (7.1%)	5 (16.1%)		1 (7.7%)	1 (7.7%)	
Length (mm^a^)	30 (27–36, 20–60)	25 (20–40, 20–90)	0.186^W^	30 (26–38, 20–60)	40 (25–55, 20–90)	0.263^W^
Width (mm^a^)	25 (20–30, 15–50)	20 (16–20, 15–50)	0.009^W^	25 (20–28, 15–50)	20 (18–28, 15–50)	0.316^W^
Area (mm^2a^)	589 (416–893, 236–2355)	393 (314–628, 236–3533)	0.050^W^	589 (408–893, 236–2355)	628 (334–1197, 235–3533)	0.738^W^

^a^Age, length, width, and area are expressed as median (interquartile ranges, ranges); area is measured by half of the maximum diameter times half of the minimum diameter multiplied by 3.14

*MBA*-*ESD* magnetic bead-assisted endoscopic submucosal dissection, *ESD* endoscopic submucosal dissection

^*F*^Fisher’s exact test, ^*W*^Whitney U-test

The details of ESD procedures in the two matched groups are shown in Table 2. Although without statistical differences, the dissection time and dissection speed were improved when using MBA-ESD (33 min vs. 40 min, *P *= 0.111; and 21 mm^2^/min vs. 16 mm^2^/min, *P *= 0.143, respectively). When performing MBA-ESD, the need of additional endoscopic instruments for dissection or hemostasis was also reduced. In general, a single magnetic bead system was enough for traction in most cases (9/13), while additional magnetic bead system may also be needed in the same (2/13) or different (2/13) sites for continual effective traction. The mean extra time for inserting and applying the magnetic bead systems was 4 ± 2 min (range 2–9). In addition, there was no collateral damage to the intestinal lumen and the specimen during the insertion and application of the magnetic bead systems in all 13 patients in the matched MBA-ESD group; system detachment also did not occur.

**Table 2:** Procedure details in the matched MBA-ESD and conventional ESD groups

	MBA-ESD (*n* = 13)	Conventional ESD (*n* = 13)	P value
Endoscopist			0.096^F^
Dr. B.H	13	9	
Others	0	4	
Endoscopic knife			0.480^F^
Dual knife or IT knife	13 (100%)	11 (84.6%)	
Dual knife + IT knife ± Hook knife	0 (0%)	2 (15.4%)	
Use of hemostatic forceps	0 (0%)	2 (15.4%)	0.480^F^
Prophylactic closure of the wound	2 (15.4%)	2 (15.4%)	1.000^F^
Use of magnetic bead system			–
Single	9 (69.2%)	–	
Double in the same site	2 (15.4%)	–	
Double in the different sites	2 (15.4%)	–	
Extra time (min^a^)	4 (2, 2–9)	–	–
Dissection time (min^b^)	33 (22–45, 18–83)	40 (30–72, 25–120)	0.111^W^
Dissection speed (mm^2^/min^a^)	21 (9, 7–34)	16 (7, 11–21)	0.143^T^
Specimen integrity	100 (100%)	–	–

The extra time for inserting and applying magnetic bead systems was included in the dissection time, and the dissection speed is measured by area/dissection time

^a^Extra time and dissection speed are expressed as mean (standard deviation, ranges)

^b^Dissection time is expressed as median (interquartile ranges, ranges)

*MBA*-*ESD* magnetic bead-assisted endoscopic submucosal dissection, *ESD* endoscopic submucosal dissection

^F^Fisher’s exact test, ^W^Whitney U-test, ^T^Student *t* test

Comparison of clinical outcomes in the two matched groups is summarized in Table 3. Although the rates of en bloc resection, R0 resection, and curative resection were similar in the two matched groups, overall complications occurred more frequently in the conventional ESD group than that in the MBA-ESD group (38.5% vs. 0%, *P *= 0.039). The most common complication in the conventional ESD group was bleeding (2 immediate and 2 delayed, respectively), and delayed perforation was also noted in one patient. Among the three patients with delayed complications, additional endoscopic intervention was required for one patient. In addition, while muscularis propria injury was noted in one (7.7%) in the conventional ESD group, there was also no such adverse event in the MBA-ESD group. All patients in the matched MBA-ESD group and the conventional ESD group showed no evidence of tumor recurrence at follow-up examination.

**Table 3:** Clinical outcomes in the matched MBA-ESD and conventional ESD groups

	MBA-ESD (*n* = 13)	Conventional ESD (*n* = 13)	*P* value
En bloc resection	13 (100%)	12 (92.3%)	1.000^F^
R0 resection	13 (100%)	12 (92.3%)	1.000^F^
Curative resection	11 (84.6%)	12 (92.3%)	1.000^F^
Overall complications	0 (0%)	5 (38.5%)	0.039^F^
Immediate bleeding	0 (0%)	2 (15.4%)	0.480^F^
Delayed bleeding	0 (0%)	2 (15.4%)	0.480^F^
Immediate perforation	0 (0%)	0 (0%)	–
Delayed perforation	0 (0%)	1 (7.7%)	1.000^F^
Muscularis injury	0 (0%)	1 (7.7%)	1.000^F^
Follow-up period (month^a^)	10 (6–13, 4–16)	6 (6–12, 3–21)	0.452^W^
Tumor recurrence	0 (0%)	0 (0%)	–

^a^Follow-up period are expressed as median (interquartile ranges, ranges)

*MBA*-*ESD* magnetic bead-assisted endoscopic submucosal dissection, *ESD* endoscopic submucosal dissection

^F^Fisher’s exact test, ^W^Whitney U-test

## Discussion

This retrospective propensity score-based matching study was conducted to compare the safety and effectiveness of MBA-ESD and conventional ESD for large superficial colorectal tumors. Regarding the safety, the rate of overall complications was significantly lower in the matched MBA-ESD group compared with the matched conventional ESD group, without additional injury to bowel lumen and the specimen during insertion and application of the magnetic bead systems. Although application of the magnetic bead systems took extra time, the comparable rates of curative resection and tumor recurrence, as well as improved dissection time and dissection speed in the matched MBA-ESD group, suggested its higher effectiveness for treating large superficial colorectal tumors.

MBA-ESD has the following advantages: (1) MBA-ESD provides additional weight traction for fully exposing submucosal layer and cutting line, more effective than tumor itself, which makes incision and coagulation more precise, and thus could reduce the risk of bleeding and perforation. Our positive results provided further evidence that traction by gravity is enough, safe, and effective for most colorectal ESD procedures [5]. (2) It is easier to adjust the weight of traction for continual effect by adding additional magnetic bead systems to the same or different sites accordingly. Magnetic force will play a role when another system is added to the same site for tumors with fibrosis [7, 8], which is also the main difference when compared with the sinker system, another device developed to facilitate colorectal ESD by gravity traction [10]. As for tumors with two or more magnetic bead systems applying to different sites, the systems may couple together because of magnetic attraction; but according to our limited experience, even though they couple together, no significant interference of the procedure would be encountered (as shown in Fig. 3), and the requirement for adding additional systems to different sites were not common (15.4%, 2/13). (3) It is simple to insert and apply the magnetic bead system, having no need for special training. Although dislodgement of the system could happen during insertion because it is just hooked on the endoclip, endoscopists can easily grasp the system again and continue further operations. (4) The cost of the magnetic bead system is lower, requiring neither expensive nor huge devices, while large magnets with high cost are frequently needed when performing magnetic anchor-guided assisted ESD (MAG-ESD) [11, 12].

A major disadvantage of the magnetic bead system is the requirement of retrieval and reintroduction of the endoscope for inserting and applying it. This may take relatively long extra time, especially for tumors in the right colon. But it seems worthwhile to spend the extra time for application of the current magnetic bead system, because that time will be paid off due to the facilitation of better submucosal exposure, ensuring lower incidence of overall complications as shown above. Through-the-scope magnetic bead system with small size may help reduce such extra time, but that means insufficient weight for traction and inadequate exposure of submucosal layer. Inspired by the application of smart self-assembling magnets for compression anastomosis in recent years [13, 14], similar through-the-scope devices with sufficient weight may be developed for facilitating ESD in the near future.

Another shortcoming of MBA-ESD is the need of patients’ position changing for adjusting the direction of traction, which is difficult especially in obese patients under anesthesia. Deployment of patient position according to tumor location in advance helps to avoid the requirement of further position changing during the procedure. Meanwhile, changing the position of patients may be replaced by using S–O clip method [15] or MAG-ESD [16]. But when using S–O clip for ESD, fracture of the spring may occur if over-stretched [17], which will increase the risk of collateral damage to the specimen and surrounding wall. As for MAG-ESD, the strong external magnetic field also can cause detachment of the internal magnet from the lesion (14.0%, 7/50) [18]. Another problem of MAG-ESD is that the coupling strength decays over distance, and thus its application in its current form is also not effective for obese patients [12]. When using external methods like modified clip-with-line method, system detachment from the tumor could also happen once the external traction is excessive or rough (13.0%, 3/23) [19]. On the contrary, there was neither system detachment nor collateral tissue damage when using MBA-ESD technique. On the one hand, a single 1.5-g magnetic bead system was enough in most cases (69.2%, 9/13), and the traction achieved is gentle; thus, tissue tearing seems to be less likely to happen. On the other hand, in cases with two or more magnetic bead systems, secure clipping of a relatively large mucosa in the edge of the tumor could help prevent tissue tearing. The recently reported internal magnetic traction device (MTD)-assisted ESD also seems a promising method for avoiding collateral tissue injury [20], in which the MTDs will disconnect once over distension occurs. However, the study involved vitro porcine stomach, and thus its role in colorectal ESD remains unclear.

Furthermore, as a technique alternative to the standard ESD, hybrid EMR-ESD is also commonly used in clinical practice. However, as reported in a meta-analysis in 2017 [21], the rates of en bloc (68.4% in 720 patients) and R0 resection (60.6% in 720 patients) of hybrid technique were significantly lower than those achieved using standard technique (91.0% and 82.9%, respectively, in 18764 patients). Thus, implementation of the hybrid technique should be of great caution, especially in those cases with submucosal fibrosis. MBA-ESD may be considered due to its effectiveness in such condition [7, 8].

This study had several limitations. First, it was a retrospective and single-center study, and the bias of selection may exist. But consecutive patients who underwent MBA-ESD and conventional ESD for superficial colorectal tumors during the study period were included; the propensity score-based matching also well balanced the baseline characteristics between the two groups. Second, the small sample size limited the statistic power of this study, and further analysis of colonic and rectal MBA-ESD was also abandoned. Large, prospective, comparative, and multi-center studies are warranted to further assess the safety and effectiveness of MBA-ESD for superficial colonic and rectal tumors, respectively. Third, we did not include patients who underwent hybrid EMR-ESD in our endoscopy center, and thus direct comparison with MBA-ESD was absent. This is mainly because lower rate of en bloc resection was commonly noted in our clinical practice. Finally, all the procedures in the MBA-ESD group were performed by a single experienced endoscopist, making it difficult to evaluate the role of MBA-ESD in other or less experienced hands.

In conclusion, MBA-ESD is feasible, safe, and effective in the treatment of large superficial colorectal tumors. Further studies are warranted to fully assess this method.
